# Cultures Through Time: Forging a Xeno-Free Future for Cell Culture-Based Virology

**DOI:** 10.3390/vaccines14060476

**Published:** 2026-05-28

**Authors:** Arvind Singh Kaulsay, Nurshariza Abdullah, Nur Amelia Azreen Adnan

**Affiliations:** 1 Malaysia Genome and Vaccine Institute, National Institutes of Biotechnology Malaysia (MGVI-NIBM), Jalan Bangi, Kajang 43000, Selangor, Malaysia; 2 Department of Biochemistry, Yong Loo Lin School of Medicine, National University of Singapore (NUS), 21 Lower Kent Ridge Road, Singapore 119077, Singapore

**Keywords:** cell culture, culture media, xeno-free, chemically defined, virology

## Abstract

As a cornerstone of modern science, cell lines are the foundational platforms for key medical advances. They enable vaccinology (through viral propagation and attenuation), gene therapy (via vector development), and biopharmaceutical production (via recombinant protein expression). Designer mammalian, avian, and insect expression systems, including Vero, MDCK, HEK293, BHK21, CHO, PER.C6, EB66, and Sf21/Sf9, have become indispensable cellular platforms, delivering enhanced biologic yields, superior genetic stability, and validated end-product biosafety. Simultaneous advances in cell culture media optimization have enabled a critical shift from serum-dependent media to serum-free, chemically defined, and xeno-free alternatives, which aim to restore compositional traceability of culture media components, reduce *potential* residual xenogeneic proteins in serum-supplemented media, and promote reproducibility even at the molecular level. This review emphasizes the far-reaching influence of cell culture systems as the expression powerhouse that sustains modern virology, whilst focusing on recent cell-engineering methods and optimization strategies in culture media that have facilitated this shift.

## 1. Introduction: Cell Cultures as Viral Expression Platforms

Cell culture represents one of the most fundamental pillars of virology and its applied disciplines. Viruses, as obligate intracellular entities, cannot exist or evolve without cellular host systems [1]. This dependency extends far beyond classical virology research; it defines the operational basis for vaccine development, gene therapy, oncolytic virotherapy, and numerous biopharmaceutical innovations. Be it mammalian, avian, or insect cell lines by origin, cell cultures provide host cell ribosomal machinery not merely for raw nucleotides but also for eukaryotic nuances such as intricate post-translational modifications (PTMs) that are vital for therapeutic efficiency, especially when intended for human use [2].

Historically, Chicken Embryonic Fibroblast (CEF) cultures, primary monkey kidney cells, and primary human fetal lung fibroblast cell strains, Wistar Institute-38 (WI-38) and Medical Research Council-5 (MRC-5), were sought-after cellular hosts of viral propagation employed to generate live-attenuated and inactivated vaccines against rubella, poliovirus, hepatitis A, rabies, and varicella [3]. However, the presence of endotoxins and adventitious agents, coupled with pronounced sourcing issues like batch-to-batch variability of livestock, ethical constraints associated with the use of fibroblasts of fetal origin, and the inherent limited life span of primary cell lines, impeded large-scale processing, highlighting the need for safer and more reliable cell-culture-based expression systems [4].

Beyond the choice of cellular hosts, the reliance on cell cultures necessitates a precisely controlled environment, which consists of the culture media capable of sustaining and optimizing complex biological processes [5,6]. A typical culture medium is composed of amino acids, vitamins, inorganic salts, and glucose, sufficient for metabolic needs and cell proliferation, whilst some culture media include animal-derived sera supplementation to sustain fastidious cell growth and viral propagation [7]. Although animal sera (Bovine-FBS) and animal-derived components (Porcine-Trypsin) have been a longstanding fundamental supplement to basal culture media and routine cell-work, the field is shifting towards *serum-independent*, *chemically defined* and *xeno-free* alternatives to curb the very same issues faced with cellular substrates because animal-derived supplements also exhibit pronounced batch-to-batch variability arising from genetic heterogeneity within source herds and breeds, poor compositional traceability, comparable sourcing and ethical constraints, and the persistent risk undefined endogenous contaminants (e.g., mycoplasma, zoonotic proteins, pyrogens, viruses, immunogenic molecules) therefore demanding the need for a “standard” across cell culture media.

This review pursues four interlocking aims: **(i)** to examine the pivotal relationship between viruses and their cellular hosts, **(ii)** to consolidate the recent advancements in cellular, media and genetic engineering that have played a part in biologics production **(iii)** to disambiguate the terms *serum-free*, *xeno-free*, and *chemically defined* in the specific context of cell-culture-based virology, and **(iv)** to offer a forward-looking perspective on the economic forces, regulatory pressures, and practical bottlenecks driving this paradigm shift.

**Figure 1:**
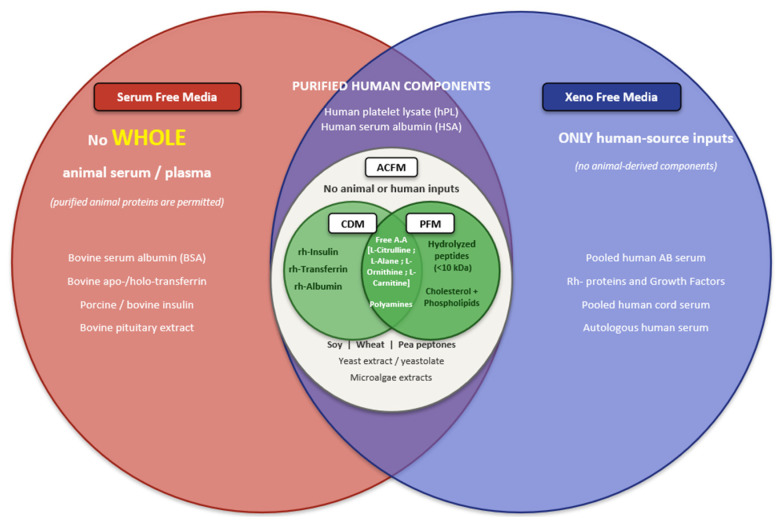
Compositional hierarchy of animal-derived component alternatives in modern cell culture media.

The terms serum-free media (**SFM**), xeno-free media (**XFM**), animal-component-free media (**ACFM**), chemically defined media (**CDM**), and protein-free media (**PFM**) are not to be used interchangeably. Whilst they are all seen as substitutes for animal-derived components, each carries a strictly distinct compositional and regulatory meaning. To clarify these overlapping yet unique relationships, Figure 1 presents a compositional Venn schematic of the principal alternatives to serum-dependent media, showing which constituents are shared across categories and which are strictly excluded from each.

Table 1 summarizes the versatile role of cell cultures across the principal facets of virology. When properly matched to their intended viral applications, mammalian, avian, insect, and engineered eukaryotic cell lines serve as adaptable expression platforms that support viral propagation, attenuation, vector assembly, and human-like post-translational modification (PTM) patterns.

## 2. Cell Cultures, Media, and Components

The art of cell culture is a versatile technique that removes prokaryotic or eukaryotic cells from their host and are then cultivated in vitro for functional and translational research [6].

In essence, cell lines and cell cultures constitute the primary biological substrates through which viral replication, attenuation, and recombinant antigen expression are operationalized, thereby enabling the reproducible production of vaccines, therapeutic viral vectors, and diagnostic biologics at commercial scales.

A complete culture system encompasses two fundamental elements: the cell line and its growth medium. Cell culture media (CCM) are classified into 2 types, Natural and Synthetic/artificial media [5,6], as elaborated in Table 2. Artificial media represent a whole market that aims to recreate a biomimetic microenvironment to sustain cellular proliferation in vitro [14,15].

Synthetic culture media are formulated and represent an engineered alternative to natural media, offering reproducibility and scalability [12], with the oldest formulation dating back to 1950, when the first chemically defined media, Medium 199, was developed to cultivate chicken embryoblasts under protein-free conditions [6]. Beyond that, cell culture media such as Basal Medium Eagle’s, Minimum Essential Medium (MEM) exist as the simplest formulations of the minimal essential amino acids for cells that do not have high metabolic requirements, whilst Dulbecco’s Modified Eagle Media (DMEM) is a modified rendition to have fourfold the concentrations of amino acids and vitamins that are present in BME [6], suitable for high metabolic demand cells.

Duly so, synthetic media as such are formulated with standard cellular necessities that include inorganic salts (NaCl, KCl, CaCl_2_, MgSO_4_, NaHCO_3_, Na_2_HPO_4_), sodium bicarbonate, and a HEPES pH buffer system, and a primary carbon source such as D-Glucose of varying concentrations as a substrate for glycolysis and energy. However, the production of synthetic media lacks labile cofactors and supplementation proteins that expedite product oxidation, which reduces shelf life. This also feeds into the notion that “one-fits-all” does not apply to culture media, because each cell line and resultant bioproducts have unique metabolic and physiological needs that must be supplemented accordingly [5].

A critical moment transpired in the 1950s, when Mr. Theodore Puck introduced fetal bovine serum (FBS) as the ideal supplement to sustain fastidious cellular growth. Since then, FBS has become synonymous with serum-dependent media (SDM) and tailored supplementation of naturally occurring growth factors across laboratories worldwide [7]. Despite extensive metabolomic/metabonomic and proteomic analyses, its composition remains incompletely defined [16]. It is known that FBS is rich in cytokines, growth factors, amino acids, and fat-soluble vitamins that are vital for cell growth, whilst containing relatively low levels of γ-globulins, which are known to exert growth-inhibitory effects [17]. This unique biochemical balance underpins the effectiveness of FBS in supporting difficult-to-culture cell lines and explains its widespread use at 2–10% (*v*/*v*) in serum-dependent systems [7]. Nevertheless, the very complexity that makes FBS indispensable also renders it an inherently undefined and variable supplement, one whose lot-to-lot inconsistency, zoonotic risk profile, and incompatibility with GMP traceability requirements have collectively driven the development of the cell engineering strategies and serum-independent culture platforms discussed in subsequent sections.

### 2.1. Types of In Vitro Expression Platforms

The selection of the right cell expression platform used for cell-culture-dependent bioprocesses is arguably the most important stage because it is the functional, genetic, and metabolic characteristics of each cell line that determine its purpose [18].

To contextualize the significance of continuous cell culture systems, Table 3 provides a comparative overview of major expression systems and their applications. By establishing these distinctions, this section sets the precedent for understanding the unique indispensability of continuous cell lines in virology and modern biotherapeutics.

#### 2.1.1. Types of Cell Culture Techniques

Cells cultured in vitro, be it mammalian, avian, or insect lines, fall under one of two categories based on their growth characteristics and morphology: Adherent and Suspension cell cultures. In terms of serving as expression platforms for vaccinology, mAb production, recombinant protein expression, and other aspects of virology, both anchorage-dependent and suspension cell lines have been used.

Adherent cultures have become ubiquitous across a wide range of biotechnological modalities [25]. For instance, adherent animal cell lines like the Baby Hamster Kidney (BHK21) cell line are renowned for their high transfection efficiency, adaptability to specific viral vectors, and consequential high viral yield for those such as the Semliki Forest Virus (SFV), Rabies, and Foot and Mouth Disease Virus (FMDV). 

Among adherent mammalian cell systems, the Human Embryonic Kidney 293 (HEK293) lineage has emerged as one of the most versatile and widely used expression platforms in modern biotechnology [5,15]. Its popularity is due to exceptional transfectability, robust protein productivity, and capacity to perform human-like post-translational modifications (PTMs) that are essential for the stability, immunogenicity, and therapeutic efficacy of recombinant biologics [6]. These characteristics have positioned HEK293 as a preferred host to produce viral vectors, recombinant proteins, vaccines, and engineered cell therapies such as chimeric antigen receptor T cells (CAR-T). Its efficiency in supporting adenoviral and adeno-associated viral vector propagation is closely linked to its innate susceptibility to viral infection, which is largely mediated by the surface expression of **αvβ1 integrin**, which acts as a primary co-receptor facilitating viral attachment and entry [25]. The critical role of **αvβ1 integrin** in adenoviral internalization was confirmed in a 2001 study, where blocking the receptor with **anti-αv** and **anti-β1** antibodies resulted in a 76% reduction in viral uptake, underscoring the mechanistic basis of HEK293’s effectiveness as a viral vector production host [26].

Likewise, the Vero cell line has become a flagship model for viral propagation in adherent systems. Genome sequencing of Vero cells found a 9 Mb deletion on chromosome 12 resulting in the complete loss of the Type-1-Interferon gene cluster (IFN-1) [6,27]. This deficiency in anti-viral cytokines like IFN-1 allows viruses to replicate, uninhibited by the host’s innate immune system. However, Vero cells and their strict adherent nature posed a problem. It was only in 2009 when Paillet et al. generated an offspring cell line, sVero, suitable for growth in suspension and serum-free environment, which was subsequently optimized for Influenza H1N1 vaccine production [27,28].

#### 2.1.2. Adherent and Suspension Cell Line Culture

Vaccine manufacturing and other large-scale bioprocesses often fail to achieve cell densities required when relying solely on adherent culture systems, as these are inherently constrained by surface area and therefore limited in scalability [25]. Consequently, these limitations have accelerated the adoption of suspension cultures, which offer scalable and cost-effective platforms for industrial bioproduction [25,29].

Unlike adherent systems, suspension cultures are not restricted by surface area, allowing cells to proliferate freely within the medium [29], which facilitates higher culture densities, in turn enhancing yield quantity. This positions suspension platforms as the preferred choice for industrial-scale manufacturing.

Nevertheless, a major challenge lies in the fact that many cell lines are not naturally suspension-competent and require extensive adaptation to anchorage-independent environments via a process known as suspension acclimation [30]. This involves serial adaptation to reduce attachment and adherent characteristics via cell passaging, which introduces selective pressure, thereby forcing the emergence of robust and evolved clones that express the suspension phenotype [30].

As an intermediate strategy for scaling up, cell growth during suspension acclimation of adherent cells can be supported with the help of microcarriers, a synthetic or natural bead-matrix that acts as a transitional platform that provides cells a 3D growth surface, therefore conforming to a *pseudo-suspension* culture system [31]. But despite their widespread use, microcarriers have not provided an ideal solution for large-scale adherent culture. Limitations such as shear stress induced by bioreactor agitation, suboptimal cell distribution, challenges in cell detachment, paired with inconsistent nutrient and oxygen transfer, have been reported [29,31]. These drawbacks highlighted the need for more effective alternatives to achieve both scalability and yield in bioprocessing.

Table 4 provides a comparative overview of adherent and suspension cell culture systems, emphasizing their defining characteristics, scalability constraints, and relevance to viral propagation and biomanufacturing.

### 2.2. Cellular and Genetic Engineering: Designer Cells

**Figure 2:**
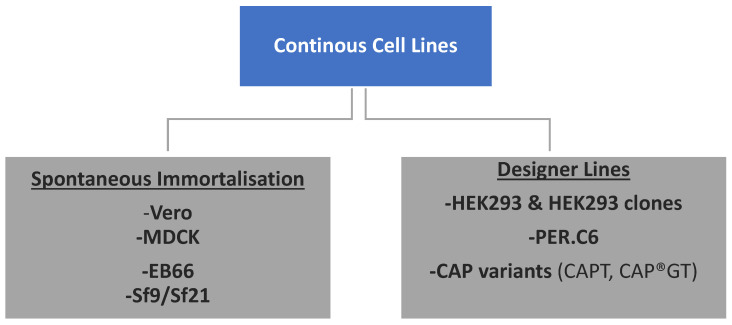
Continuous cell lines were differentiated based on how immortalization was acquired.

Advances in cell engineering have subsequently enabled the development of “designer” cell lines tailored for specific bioprocesses. Genetic manipulation strategies, including targeted genome editing with CRISPR-Cas9, have facilitated the selection and introduction of desired traits, ultimately generating cell systems optimized for defined industrial applications. Figure 2 is an illustration of continuous cell lines that achieved immortality via spontaneous and induced means.

As established, CHO and HEK293 continuous cell lines are the chosen workhorses for mimicking human-like PTMs for biotherapeutic production. but are unable to reproduce the exact human PTM landscape. Especially in their wild-type form, both cell types generate non-human glycan structures such as N-glycolylneuraminic acid **(Neu5Gc)** and **α-Gal (galactose-α1,3-galactose)** epitopes [34]. This induces unprecedented immunogenic responses towards biologics intended for human use because Neu5Gc and α-Gal are considered human xenoantigens [35]. For certain biologics, these PTMs are indispensable: mAbs rely on precise Fc glycosylation to engage immune effector functions, clotting factors such as Factor VIII require correct glycosylation for stability and therapeutic efficacy [36], whilst viral glycoproteins such as the SARS-CoV-2 spike protein depend on authentic glycan shielding to preserve their native conformation and antigenicity [37,38].

This opportunity gave rise to the concept and reality of “designer” mammalian expression systems, spearheaded by the CHO FUT8−/− [fucosyltransferase knockout] cell line [39]. This glycoengineered subset of CHO cell lines served as a cell substrate for **Mogamulizumab**, the first-ever defucosylated, glycoengineered mAb for the treatment of CCR4-positive T cell leukemia or lymphoma. This form of immunomodulation produced mAbs with defucosylated Fc regions, which enhanced antibody-dependent cellular cytotoxicity (ADCC) activity and contributed to the Mogamulizumab mechanism of action [35].

Referring to Table 5, engineered derivatives of the parental HEK293 lineage, like HEK293T, HEK293E, and 293F, were developed to enhance viral vector production, gene expression, or culture scalability [40]. HEK293T cells were created through stable expression of the **Simian Virus 40 (SV40) large T antigen**, which binds and inactivates tumor suppressors p53 and Rb, promotes SV40 replication and episomal amplification of plasmids containing the SV40 origin of replication (*ori*) [8,40]. This modification makes HEK293T the ideal host for transient plasmid transfections of LV and AAV vectors, a concept employed in the development of Kymriah^®^ for B-cell precursor acute lymphoblastic leukemia [8]. In contrast, HEK293E cells were engineered to express the **Epstein–Barr virus nuclear antigen-1 (EBNA1)** [40], which supports episomal replication of vectors carrying the Epstein–Barr virus origin of replication (EBVoriP), optimized for transient gene expression (TGE), perfect for applications like rZIKV NS1-His expressing 293E cells for Zika diagnosis and surveillance purposes [24].

The engineering of HEK293T as a viral vector production platform has advanced considerably beyond the SV40 T antigen modification. Due to the increase in viral vector demand employed in therapies such as Kymriah, Breyanzi (to treat B-cell lymphoma), and Abecma (to treat Multiple Myeloma), LV biosynthesis is often insufficient, and the production of viral vectors is costly to scale [46]. To address this restriction, a genome-wide CRISPR screen employing cytosine base editors across 17,501 genes in HEK293T cells identified nine genes that actively limit lentiviral vector packaging and formation, whereby knockout of the LDAH (lipid droplet-associated hydrolase) gene itself led to a 6.63-fold increase in LV titers, whilst triple gene KO of **GBP3**, **BPIFC**, and **LDAH** saw an 8.33-fold boost. The study conducted by Zhang et al. in 2024 demonstrates that host cell restriction landscape represents a largely untapped lever for vaccine and vector yield optimization [47].

Another promising expression platform is the CAP^®^ cell line (CEVEC’s Amniocyte Production). Genetically altered, this nonmalignant suspension cell line of immortalized amniocytes serves as a factory for recombinant protein production, with excellent biologic activity and therapeutic efficacy as a result of authentic human PTMs [48]. CAP cells grow to high densities in serum-free suspension culture and have demonstrated robust performance across virological applications, including influenza virus propagation, where hemagglutination titers exceeding 3.0 log_10_ HA units and high cell-specific virus productivity have been reported. In parallel, CAP-derived systems such as SV40, consisting of CAP-T cells, yielded ~2.5 fold higher plasmid copy numbers per cell than comparable HEK293T cultures, whilst CAP^®^GT achieved cell densities up to $2\times 10^{7}$ cells/mL in all common bioreactor formats, thereby significantly outperforming HEK293 as AV, LV, and AAV vector platforms [48].

A key limitation of continuous adherent cell lines is their intrinsic dependence on surface attachment and scalability constraints. Addressing this bottleneck, a study titled “Comparison of Vero and MDCK cell lines transfected with human SIAT7E gene for conversion to suspension culture” demonstrated that transfection with human SIAT7E successfully converted adherent Vero and MDCK cells into suspension cultures [49]. Located on chromosome 1, the human **SIAT7E** gene encodes for the **SIAT7E** enzyme, which catalyzes the biosynthesis of **ganglioside GD1alpha** from GM1b in the brain, thereby modulating neuronal communication within humans [49]. With the use of differential gene expression analysis, the **SIAT7E** gene was found to be one of the genes that has a profound influence on cell adhesion and cellular interactions due to its intrinsic sialyltransferase activity. This study hypothesized that upregulating the transcription of the SIAT7E gene conferred a reduced degree of cell adhesion. Experimental validation, performed on MDCK and Vero cells, clarified this phenomenon: cell lines overexpressing SIAT7E developed a pronounced negative charge on the cell surface in comparison to parental cells [49]. Heightened negative charge subsequently led to increased electrostatic repulsion between cells, consequently facilitating their ability to grow in suspension [49,50]. Conceptually similar results were obtained by overexpressing sialyltransferases **ST6GAL1** and **ST3GAL6** in **B4GALNT3/4-KO HEK293** cells to achieve better Factor VII-albumin sialylation and intended pharmacokinetics [51].

Notably, modern non-mammalian and engineered human substrates such as Sf9/Sf21, EB66^®^, and embryonic retinal PER.C6^®^ cells were established, propagated, and optimized from inception under SF, XF, and CD conditions, thereby circumventing the laborious adaptation campaigns that legacy mammalian platforms (HEK293, Vero, CHO, MDCK) required to transition from serum-dependent platforms.

Altogether, the trajectory of cell engineering has moved beyond single-trait optimization to the creation of cellular expression systems that combine human-like glycosylation patterns, suspension growth, and serum-free adaptability, thereby overcoming previous limitations and reducing the risk of aberrant or immunogenic bioproducts. By replacing and eliminating the need for undefined animal components in culture media, these engineered systems align with Good Manufacturing Practice (GMP) standards and address the increasingly strict regulatory requirements for safety, consistency, and scalability in biomanufacturing [18].

### 2.3. Transitioning Towards Xeno-Free Alternatives

As stated earlier in Section 2, the growth media of a cell culture are arguably the most important aspect. Exemplified in Table 6, Synthetic media can be categorized based on their **serum supplementation**, **composition,** and the **biological origin** of components, ranging from animal-derived to fully xeno-free and chemically defined formulations.

Building upon the principles of Good Manufacturing Practices and the clear advantages of traceable, well-defined components in biologics, cell biologists are keenly exploring the replacement of animal-derived substances in CCM and its supplements.

Fetal Bovine Serum (FBS) and other xeno-derived sera play a pivotal role in cell media by supplementing a rich, albeit undefined, cocktail of essential nutrients, growth factors, hormones, and attachment proteins when supplemented into basal growth media. Functionally, serum aids cell growth, metabolism, and functions as a protective barrier from proteolytic enzymes such as Trypsin, thereby preserving cell integrity during routine culture manipulations and passaging.

As early as the 1970s, the undefined nature and variability of animal sera had already been recognized as major limitations in reproducible bioprocessing. Over subsequent decades, concerns regarding zoonotic diseases like the Bovine Spongiform Encephalopathy (BSE), adventitious viruses and pathogens, and ethical issues surrounding bovine and porcine-derived components led governing authorities such as the WHO, FDA, EMA, ICH, and national regulatory agencies to mandate rigorous screening of serum-containing media while simultaneously encouraging the adoption of xeno-free culture systems [52]. This dual approach reflects the need to ensure immediate biosafety in existing processes and the long-term goal of fully transitioning to defined, GMP-compliant substrates for human therapeutics.

In addition to that, protein concentration found in media supplemented with 10% sera has a range of 6200–10,000 mg/L total protein, whilst the concentration for a defined recombinant protein produced in mammalian cells does not usually exceed 1000 mg/L [15]. This discrepancy poses major challenges in the downstream production of protein-based drugs and recombinant proteins, predominantly seen during the manufacture of recombinant human IgG, where bovine IgG co-binds Protein A resin (method of Ab binding chromatography) via its conserved Fc domain and co-elutes with recombinant human IgG, thereby creating a downstream processing bottleneck that requires additional orthogonal separation steps [53].

Due to this, serum-free, animal-protein-free, and chemically defined media emerged as regulatory-preferred platforms for GMP-compliant and xeno-free cell culture [5,15]. While widespread adoption remains limited by technical and economic constraints, cell line engineering has advanced in parallel with serum-free media [9], with established workhorses such as Vero, MDCK, and HEK293 variants now thriving under serum-independent conditions [11,28,51].

For instance, research by Park et al. highlights the potential of marine microalgae extracts as effective alternatives to serum supplementation in cell culture. Specifically, extracts from *Dunaliella salina* (DS) and *Spirulina platensis* (SP) significantly enhanced proliferation in MDCK and Vero cells, with DS achieving proliferation rates of 149.56% and 195.50% compared to conventional serum-free media [28]. In addition to supporting higher cell viability, these extracts also increased superoxide dismutase activity, providing antioxidative protection that reduces oxidative stress within cultured cells [28]. By demonstrating that phytochemical-based supplements from microalgae can simultaneously promote growth and protect against oxidative damage, this study strengthens the case for reducing the reliance on FBS.

Furthermore, a product study conducted by MP Biomedicals evaluated the proliferation dynamics of Vero cells cultured in FBS-supplemented and FastGro™ serum-free media over a six-day period. With an initial seeding density of 20,000 cells/cm^2^, cultures maintained in Williams Medium E supplemented with 10% FastGro™ achieved a final cell density of 197,000 cells/cm^2^, compared to 184,000 cells/cm^2^ in the 10% FBS control [54]. This represents a 7% increase in both total cell yield and the cell multiplication index when using chemically defined media like FastGro™. 

These findings underscore the remarkable efficiency of serum-free formulations as viable, possibly superior, alternatives to serum-based systems. As virology increasingly intersects with regulated biomanufacturing and human therapeutics, the authors believe such media innovations represent a critical step toward eliminating animal-derived variability.

Moreover, the widespread adoption of serum-free media in bioprocessing has rendered conventional trypsin redundant. Porcine-derived trypsin is a serine-protease enzyme that hydrolyses peptide bonds within proteins and between amino acids, exclusively at the C-terminal to Lysine and Arginine. By this mechanism, Trypsin is used as a cell-dissociation agent to dislodge cells prior to subculturing, cell counting, cryopreservation, and any culture manipulation that requires the adherent cell to be “free” from its vessel. This step has hence earned the name, trypsinization.

However, in the absence of FBS, which contains protease inhibitors such as **α1-antitrypsin** and **α2-macroglobulin** capable of inactivating residual trypsin activity and buffering its cytotoxic effects, serum-free media lacks such protective components [55]. Consequently, there is a growing reliance on animal-origin-free media and recombinant trypsin alternatives that provide gentler, more consistent detachment without compromising cell integrity or compatibility with chemically defined and animal-free systems.

TrypLE™ is one such alternative to conventional Trypsin. TrypLE™ is a recombinant fungal trypsin-like protease, which has proven effective at dissociating many different adherent mammalian cell lines. In a comparative study, Tsuji et al. reported that while trypsin reduced the expression of several mesenchymal stem cell surface antigens, TrypLE™ preserved surface-antigen integrity, highlighting its gentle action during cell dissociation compared with the traditional enzyme [56]. Similarly, Accutase™ is another mild-acting, xeno-free dissociation reagent composed of proteolytic and collagenolytic enzymes [30]. Both products serve as direct replacements for conventional trypsin, with practical advantages such as eliminating the requirement for serum-mediated inactivation after passaging. By bypassing this step, TrypLE™ and Accutase™ enable gentler handling of cells and improve the overall efficiency of routine cell culture compared to traditional trypsin.

### 2.4. Analyzing Future Prospects: Applications and Caveats of Serum-Free Media and Xeno-Free Substitutes

The field of cell biology and cell engineering is undergoing a critical inflection point marked by the growing adoption of xeno-free systems. This transition is not merely a preference, but an inevitable trajectory driven by the confluence of regulatory scrutiny, safety standards, ethical sourcing, and the critical need for scalability.

The core principle is that while technical adaptation challenges persist, particularly concerning the necessary modification of known cell lines, the industry’s commitment to this shift is affirmed by the high-growth trajectory of the defined media market, which has grossed USD 1.9 billion as of 2024 and is projected to reach USD 4.05 billion by 2030 for serum-free formulations alone [57]. 

Significant advancements have been made to improve the performance of serum-free media in bioproduction to resolve the lack of endogenous transport and protective proteins found in serum. In a study performed by Novo Nordisk Pharmatec, recombinant insulin was used to boost influenza virus production via the HEK293SF-3F6 cell platform. Said to stimulate anti-apoptotic and mitogenic pathways, insulin-supplemented cultures showed increased viral titers of ~1.7-fold. During infection, the influenza virus activates the PI3K/Akt pathway to facilitate viral entry and promote its replication and proliferation within host cells. In the presence of insulin, Akt phosphorylation was further increased, suggesting that insulin enhances influenza virus production by further activating the PI3K/Akt pathway [58].

Beyond other benefits, implementing xeno-free alternatives in biopharmaceutical production of mAbs, vaccines, cell therapies, immunotherapies, and other human bioproducts prevents the risk of xenoimmunization, a recipient immune response triggered due to residual non-human, animal-derived components from the cell culture process. Minimizing this risk is imperative for advanced therapies like CAR-T, cell and gene therapy (CGT) modalities, and stem cell biologics, as even trace amounts of foreign matter can drastically alter pharmacodynamics/kinetics, safety, and overall efficiency of the therapy.

Notwithstanding the trajectory of CD and XF media, serum-dependent systems are expected to retain near-term relevance as certain cell lines exhibit suboptimal proliferation and subsequent downstream bioprocesses when deprived of animal-derived growth factors within sera [59]. Furthermore, certain commercially available CDM formulations have been documented to cause cell aggregation-driven culture failure. The authors observed this directly during sequential adaptation of HEK293 cells from 10% to 0% FBS across two chemically defined media formulations. Cells maintained in one CDM formulation developed pronounced aggregation and clump formation visible to the naked eye, characteristic of aberrant suspension behavior rather than healthy monolayer cell growth, whilst the very same cell line that successfully adapted to the other CDM formulation showed healthy cell morphology and confluence throughout the adaptation protocol. Comparable medium-specific aggregation failure in CHO platforms was independently corroborated by Reinhart et al. [59] in 2015, reinforcing that CDM incompatibility is a reproducible, cell line and medium-specific phenomenon rather than an isolated observation, when not optimized for.

The adaptation protocol that ultimately proved effective involved centrifugation-based passaging in lieu of enzymatic dissociation: at each passage, cells were pelleted by centrifugation, the supernatant discarded, the cell pellet resuspended in fresh HEK293 CDM, and then cultured in T25’s The authors propose this centrifugation-resuspension passaging strategy as a practical method to obtain a pure cell pellet by stripping the cell suspension of FBS, thereby facilitating sequential adaptation to select serum-independent systems.

Furthermore, the complexity and specificity of SFM and CDM formulations, while offering benefits in terms of consistency and safety, inherently limit their cross-usability across different cell lines and processes. Consequently, the realization of a truly universal, “one-fits-all” xeno-free medium remains an elusive goal, or at least for now.

While an immediate, wholesale shift to xeno-free systems in cell-culture-based biotherapeutics might not seem feasible, animal-free and chemically defined growth media have become the de facto standard for the expansion of human Mesenchymal Stem/Stromal Cells (hMSCs) for regenerative medicine applications [60]. As highlighted by Bui et al., the benefit of transitioning to xeno-free systems extends beyond the replacement of basal media; it also encompasses the shift toward xeno-free cell dissociation reagents, which is particularly vital for hMSC cultivation, as the preservation of specific surface antigens like CD73, CD90, and CD105 is an essential identifier of hMSCs according to the International Society of Cellular Therapy. Consequently, replacing animal-derived trypsin with gentler, xeno-free dissociation reagents is crucial to maintaining the phenotypic integrity and therapeutic potential of hMSCs [60].

Intriguingly, the demand for xeno-free alternatives has extended beyond biopharmaceuticals into industries pursuing cruelty-free or “slaughter-free” products such as cultivated meat (CM), where reliance on FBS presents both philosophical and commercial contradictions. Because CM production requires industrial-scale volumes of culture media, the high cost of recombinant growth factors represents a major economic barrier. To address this, some platforms have adopted in-house recombinant expression systems, including *Escherichia coli*, to produce growth factor orthologs at substantially reduced costs (reported as low as $0.82 per liter of media) [61]. These advances are not isolated to food biotechnology; they illustrate scalable strategies for eliminating animal-derived inputs while maintaining proliferative capacity. Such approaches are directly relevant to virus-related cell substrates and vaccine manufacturing, suggesting that large-scale biomanufacturers must evaluate internal production capabilities to accelerate the transition towards a chemically defined and xeno-free future [61].

Beyond compliance and cost control, replacing serum for recombinant proteins directly augments viral productivity. Alfano et al. demonstrated this by developing OptiVERO, a chemically defined, blood-free medium formulated around two recombinant human proteins: **Cellastim S** (recombinant human serum albumin) and **Optiferrin** (recombinant human transferrin), supplemented with recombinant human epidermal growth factor of non-mammalian origin. Comparison studies between OptiVero, EMEM-10% FBS, and VP-SFM (plant hydrolysate SFM) showed cells cultured in OptiVERO had 50–70% higher cell density than those in FBS and VP-SFM controls from Day 2 to Day 9 post-seeding, whilst supporting robust viral production, which was studied by quantifying DenV-2, ZikV, and Ebola∆VP30 titers [62].

## 3. Conclusions: Embracing Change

The evidence reviewed here makes one thing unambiguous: the co-evolution of engineered host cell systems and chemically defined media is not a preference of the research community; it is a biological and regulatory necessity. However, several structural constraints remain unresolved and warrant candid discussion.

The medium-incompatibility problem remains unsolved. As the authors observed directly during sequential HEK293 adaptation, and as corroborated by Reinhart et al., chemically defined media can induce profound, aggregation-driven culture failure. Until validated strategies for predicting CDM compatibility from cell line transcriptomic and metabolomic profiles are established, this bottleneck will continue to inflate development timelines and deter adoption in resource-constrained settings.

Furthermore, the economic barrier to SFM and CDM adoption remains the most structurally entrenched obstacle. SFM used in cell cultivation is expensive, contributing to at least 50% [63] of variable operating costs, whilst over 95% of production costs are attributed to culture medium, due to the very high prices for recombinant growth factors and other components such as serum albumin. The emergence of microbial cell-free protein expression platforms, including *E. coli*-based and BY2-based systems capable of yielding approximately 3 g/L of recombinant growth factors, offers a credible route to cost reduction. Still, current applications of recombinant growth factors are primarily in the biopharmaceutical industry and for academic research, where smaller quantities suffice, and cost constraints are less stringent [64]. A manufacturing paradigm dependent on expensive, chemically defined inputs risks replicating the access disparities witnessed during COVID-19, where fewer than 2% of people in the poorest nations had received a vaccine [65].

Fiscal restraints do not stop there when the most capable expression platforms discussed in this review are not freely available biological tools. They are cataloged, tiered, and priced commercial products. According to ATCC, a standard research-grade frozen vial of parental HEK293, Vero (CCL-81), CHO-K1, and Sf9 costs $577 (≅RM 2285) per vial, sufficient for following optimized bioprocesses and routine laboratory functions of cell maintenance, protein expression, and viral seeding. In the instance a manufacturer sought cGMP-grade Vero for actual vaccine production, it would cost upward of $13,000 (≅RM 52,000), before a single regulatory qualification step has begun. Truly optimized, flagship, validated platforms like PER.C6^®^, owned by Janssen Vaccines and Prevention B.V. and licensed to MNC’s like Merck, GSK, Novartis, Pfizer, and EB66^®^, Valneva’s proprietary duck embryonic stem cell platform with over 35 commercial license agreements worldwide, are not available on any catalog at any price. Access to these Intellectual Property-gated platforms requires direct negotiation of research licenses, annual maintenance payments, and product royalties, with financial terms that are never publicly disclosed.

Creative solutions to these constraints are emerging on three fronts. The most promising near-term strategy is the integration of multi-omics profiling with AI-guided media optimization. Bayesian optimization-based frameworks have demonstrated the capacity to identify and develop superior media compositions using three to thirty times fewer experiments than conventional Design of Experiments approaches [66].

The author suggests another possible improvement, i.e., rather than engineering media to compensate for serum-dependency, CRISPR-based approaches can engineer serum-independence directly into the cell. Gene-KO to facilitate viral vector production showed us that the host cell genome is a largely untapped lever. Gene ontology mapping, identifying and disrupting genetic programs that anchor cell lines to serum-dependent survival, would produce expression platforms that are robustly defined-media compatible by design, rather than by empirical adaptation.

The last front that would aid adoption of animal-derived cell culture alternatives would necessitate regulatory harmony between the FDA, EMA, and regional agencies on xeno-free raw material standards to further reduce the compliance burden that slows adoption. It was the FDA’s own April 2024 guidance “Considerations for the Use of Human- and Animal-Derived Materials in the Manufacture of Cell and Gene Therapy and Tissue-Engineered Medical Products” [67] that revealed the scale of this problem, albeit indirectly: the guidance addressed animal-derived material qualification at length but contained only a footnote-level acknowledgement that read “serum-free medium and supplements may still contain human or animal components”. Regulatory analysts like Blake Bergam and Sara Mills of the Dark Horse Consulting Group [68] stated that this left the broader terminology landscape entirely unaddressed, and that “serum-free,” “protein-free,” and “xeno-free” terminologies carry no enforceable regulatory meaning in the absence of FDA-defined standards which will only hinder the adoption of chemically defined systems regardless of how compelling the scientific case for them becomes.

In sum, the evolution of cell culture systems in virology is best understood as a unified engineering response to sustaining the biological needs of virus-producing cells with increasing precision, safety, and scalability, while eliminating the undefined inputs that have historically made these systems work.

The tools to complete this transition now exist: glycoengineered cell lines, AI-guided media design, CRISPR-enabled serum independence, and cost-reduced recombinant protein production. What remains is a policy choice, whether the optimized cell line platforms and chemically defined media formulations that make this transition possible are treated as shared scientific infrastructure for global health, or retained as proprietary commercial assets accessible only to those who can afford them.

## Figures and Tables

**Table 1:** Cell culture platforms supporting viral expression, vector production, and therapeutic virology applications.

Application	Viral Vector	Host Cell System	Importance of Expression Platform	References
Vaccinology	IFv, DengV, NoV, SARS-CoV-2, and potentially any viral pathogen that has been identified/sequenced	CEF Madin Darby Canine Kidney (MDCK) HEK293/293F MRC5 Vero PER.C6	Viral virulence decreases with each cell passage, thereby obtaining inactive/live attenuated vaccines. Certain mammalian cell lines are preferred as they perform appropriate PTMs and protein folding, which gives rise to Subunit and Epitope-based vaccines. Vero used as a cell–substrate for CoronaVac, MDCK for live-attenuated Influenza virus [Flucelvax], or PER.C6 for viral-vector based SARS-CoV-2 vaccine [JCOVDEN].	[8]
Gene therapy	AAV, ADV, Lenti/Retrovirus based on the desired length of gene expression (transient/stable expression)	HEK293/293T MDCK BHK21	HEK293 (T) cells are favored for ADV and AAV vector production due to their efficient transfection and consistent viral titer yield. FDA-approved AAV-RPE65 gene therapy using the HEK293 cell line to treat inherited retinal dystrophy [Luxturna^®^] since 2017.	[9]
CAR-T Cell therapy	HIV-1-based Lentivirus and γ-Retroviral vectors	HEK293/293T/293F	Viral plasmids are transfected into the host cell for assembly into viral vectors, which are then used to genetically modify T cells for CAR-T therapy. HEK293T cell lines, expressing SV40 large T antigen, boost viral vector titers by enhancing replication of transfected plasmids containing the SV40 origin of replication [Ex. FDA approved Kymriah^®^ using HEK293 assembled LV for CD19-directed CAR-T cell therapy]	[10,11]
Oncolytic therapy	MG1-derived Maraba virus, Recombinant Poliovirus (PVSRIPO), Augmented HSV-1, VSV-NDV	EB66 suspension quail cells Vero BHK21 HEK293 A549 lung carcinoma epithelial cell line	Vero cells are interferon-deficient, making them highly susceptible to infection due to defective antiviral defenses. Culturing oncolytic viruses like ADV and HSV requires continuous mammalian/avian cell lines to retain viral tropism, infectivity, and viral potency. A549 lung adenocarcinoma cell line is used to screen cytolytic activity of oncolytic viruses and serves as a platform to study host–pathogen interactions, i.e., COVID-19, IfV.	[12,13]

**Table 2:** Overview of natural and synthetic cell culture media categories.

Media Category	Subtype	Representative Examples
Natural Media	Biological fluids	Serum, plasma, lymph, amniotic fluid, human cord serum
	Crude tissue extracts	Liver, spleen, embryo extracts (bovine, chick)
Synthetic Media	Basal	MEM, EMEM, DMEM
	Complex	RPMI-1640, IMDM, Ham’s F-12
	Serum-dependent	DMEM + FBS, RPMI + FBS
	Serum-free	Hybridoma-SFM, VP-SFM, HEK293SFM
	Chemically defined	Medium 199, CD-CHO, CD-293, 4Cell^®^HEK
	Xeno-free	XF-293, XF-Vero

**Table 3:** Comparative overview of common expression systems.

Expression System	Host Systems	Key Advantages	Limitations/Challenges	Notable Applications	References
Prokaryotic	*Escherichia coli* *Bacillus subtilis*	Rapid growth and high yield. Inexpensive media and simple scale-up. Generally Recognized as Safe (GRAS)	Lacks replicability of human-like PTM ∴ misfolded, insoluble proteins that accumulate as inclusion bodies.	Recombinant protein production (Insulin, growth factors).	[19,20]
Yeast	*Saccharomyces cerevisiae* *Pichia pastoris*	Performs basic PTMs (glycosylation, disulfide bonds). High cell density cultivation.	Hypermannosylation patterns differ from humans. Lower yield of complex proteins.	Recombinant subunit vaccines (HBsAg for Hepatitis B).	[21]
Insect Cell–Baculovirus System	*Spodoptera frugiperda* (Sf9, Sf21) *Trichoplusia ni* (High Five)	Handles complex eukaryotic PTMs. High-level expression via baculovirus vectors. Scalable suspension culture.	Costlier than microbial systems. Slower growth rate. Glycosylation patterns differ slightly from mammalian cells.	Recombinant subunit vaccines (Flublok^®^ for influenza, NVX-CoV2373 COVID-19 vaccine). VLP and protein complex production.	[22,23]
Mammalian Cell Lines	CHO HEK293 Vero MDCK	Accurate human-like PTMs and good protein quality. Regulatory precedence for biologics.	Slow growth and expensive media. Susceptible to viral contamination. Scale-up complexity.	Monoclonal antibodies (mAbs). Viral vector vaccines (AstraZeneca, J&J COVID-19).	[24]
Plant-Based	*Nicotiana* *benthamiana* *Arabidopsis* *thaliana*	Low human pathogen risk. Low production cost. Rapid scalability.	Complex downstream purification of plant polyphenols. Glycosylation and bioactivity issues.	Tri-chimeric mAbs for Ebola (e.g., ZMapp^®^)	[23]

**Table 4:** Key differences between adherent and suspension cell culture systems.

Parameters	Culture Types		References
	Adherent	Suspension (Anchorage Independent)	
Definition	Anchorage-dependent cells require a solid surface to grow as a monolayer.	Cultivation of cells does not require attachment to a surface, as cells float and proliferate freely.	[32]
Equipment required	T-flasks, microwell plates, roller bottles	Bioreactors, Erlenmeyer shaker flasks, wave bags, and microcarriers.	[9]
Rate-limiting steps	Surface area for cell growth is often a rate-limiting step because overconfluence will increase intercellular competition for media and growth factors. Cell detachment from culture flasks will hinder cell growth.	Concentration of cells within the culture media creates a rate-limiting step. It is essential to monitor the growth rates in suspension cultures over time. Shear stress in stirred systems, i.e., bioreactors, will incur cell death.	[32]
Upstream Scalability	Poor scalability due to limited growth area BUT makes for good cellular study models to observe cell-to-cell adhesions and polarity.	Scalable for large-batch, high-density cultures, which are necessary in fields like recombinant protein production and viral vaccinology.	[31]
Applications	BHK21 cells grown on a microcarrier system increased the capacity of viral yield for the inactivated rabies candidate vaccine YU BHK Rabivak.	Glutamine synthetase-KO CHO cell line used to cultivate recombinant mAb therapies like Rituximab. Inactivated Influenza vaccine [Flucelvax^®^] made using MDCK 33016-PF suspension-adapted cell line as substrate.	[32,33]
Examples	HEK293, Vero, MDCK, MRC5, WI38, CEF, BHK21.	HEK293-F, EB66, SF9/21, MDCK-S.	

**Table 5:** Commonly used continuous lines, methods to attain natural or induced genetic changes, and applications.

Cell Line	Growth Requirements	Method of Immortalization	Applications	References
HEK293T	Adherent/Suspension adapted.	Stable transfection of HEK293 with a plasmid encoding SV40 T antigen.	Utilized in suspension culture for high-titer rAAV production.	[8,9]
HEK293F	Suspension	Subclones of suspension-adapted HEK293 cells were isolated and cloned.	Used for large-scale production of recombinant proteins like rFVIII (NUWIQ^®^)	[8,9]
HEK293E	Suspension	Stable transfection with plasmids encoding viral EBNA1.	Widely used in transgene expression due to EBNA1, which enhances the cell’s ability for episomal replication of oriP-harboring plasmids.	[8,9]
HEK293S	Suspension adapted.	Serial passages in modified MEM.	Glycoengineered variants devoid of *N*-acetylglucosaminyltransferase I (KO-GnTI^−^) are used for high-throughput production of deglycosylatable glycoproteins, excellent for crystallography.	[41]
Sf9/Sf21	Adapted to adherent and non-adherent conditions, does not require CO_2_ supplementation and can thrive in serum-free media.	*Spodoptera frugiperda* 21 was the original line isolated, while Sf9 was clonally derived for superior suspension growth and stability.	Extensively used in the Baculovirus Expression Vector System (BEVS) for high-yield production of recombinant proteins, virus-like particles (VLPs), and vaccines (Cervarix^®^). Subset cell line *expres*SF+ used to make FluBlok^®^-recombinant hemagglutinin influenza vaccine.	[18,42]
PER.C6^®^	Human embryonic retinal cells, suspension-based.	Created by transfecting retinal cells with a plasmid encoding only E1A and E1B, flanked by known sequences, under a CMV promoter.	PER.C6^®^ used as a production platform for Janssen’s Ad26.COV2. S recombinant vaccine.	[43]
CHO-S	Chinese Hamster Ovary (suspension).	Long-term adaptation to serum-free suspension.	Gold standard for mAb production, Fc-fusion proteins, and therapeutic clotting factors due to superior genetic stability and ability to perform human-like PTMs.	[44]
EB66^®^	Duck embryonic stem cells, suspension-based.	Relied on the effects of natural selection and clonal isolation rather than direct genetic manipulation.	EB66^®^ is a highly permissive platform for MVA-based vectors, sustaining robust transgene expression, superior viral titer compared to CEF, and rapid scalability (100 Lof *rMVA* in 3 weeks).	[45]

[SV40—Simian Virus 40; EBNA1—Epstein–Barr Virus Nuclear Antigen 1; oriP—Origin of Replication; rFVIII—recombinant factor 8; MEM—Minimal Eagles Media; mAb—monoclonal Antibody; CMV—cytomegalovirus; PTM—post-translational modifications; MVA—modified vaccinia virus].

**Table 6:** A comparison of commonly used synthetic growth media.

Media Type	Definition	Pros	Cons	References
Serum-Dependent Media (SDM)	Basal medium supplemented with serum (e.g., Fetal Bovine Serum—FBS). Contains undefined hormones, growth factors (GFs), and proteins.	Historically established and widely compatible with many cell lines. Low initial raw material cost compared to defined alternatives.	High lot-to-lot biological variability. High regulatory risk for BSE/viral contamination, requiring extensive and costly testing. Undefined components can interfere with downstream purification.	[5]
Serum-Free Media (SFM)	Does not contain whole serum but may contain serum-derived components (e.g., BSA) and protein hydrolysates (e.g., from soy or wheat).	Eliminates the fundamental risks and high variability associated with serum. Improves regulatory compliance over SDM.	A lack of chemical definition when using hydrolysates can still lead to process variability and complicated root-cause analysis. Cell line adaptation is a major challenge. Adapted cells may show accentuated sensitivity to routine manipulations like centrifugation and trypsinization.	[5]
Xeno-Free Media (XFM)	Often uses human-derived supplements like human serum (huS), human platelet lysate (hPL), or human serum albumin (HSA). Animal-derived components are strictly prohibited as supplementation.	Eliminates non-human animal risks (e.g., TSE, non-human animal viruses). Suitable for cells intended for human use (e.g., Cell Therapy, ATMPs).	Human-derived components (huS, hPL) still carry a risk of human pathogen transmission. Requires complex GMP sourcing, testing, and regulatory qualification for donor material. Limited universal applicability.	[5]
Chemically Defined Media (CDM)	Requires that all of the components be identified, as well as their concentrations. Animal-Free (AF) is a common synonym. Must be completely free from serum, animal-derived or human, and albumin-free.	Gold standard for consistency; provides maximum batch-to-batch reproducibility. Highest compliance with GMP and regulatory guidelines (FDA, EMA).	The highest raw material cost is driven primarily by expensive recombinant Growth Factors (GFs) and recombinant proteins. Initial capital constraints can bottleneck usability in start-ups or academic labs.	[5]

## Data Availability

No new data were created or analyzed in this study. Data sharing is not applicable to this article.
